# Exosomes and Cancer Stem Cells in Cancer Immunity: Current Reports and Future Directions

**DOI:** 10.3390/vaccines9050441

**Published:** 2021-05-01

**Authors:** Na-Kyeong Lee, Vinoth Kumar Kothandan, Sangeetha Kothandan, Youngro Byun, Seung-Rim Hwang

**Affiliations:** 1 College of Pharmacy, Seoul National University, 1 Gwanak-ro, Gwanak-gu, Seoul 08826, Korea; enklee8@gmail.com (N.-K.L.); yrbyun@snu.ac.kr (Y.B.); 2 Department of Biomedical Sciences, Graduate School, Chosun University, 309 Pilmun-daero, Dong-gu, Gwangju 61452, Korea; vino2787@ymail.com; 3 Department of Biotechnology, Saveetha School of Engineering, Saveetha Institute of Medical and Technical Sciences, Chennai 600073, India; sangeethak.sse@saveetha.com; 4 College of Pharmacy, Chosun University, 309 Pilmun-daero, Dong-gu, Gwangju 61452, Korea

**Keywords:** exosome, cancer stem cell, cancer stem cell-derived exosome, immunotherapy, exosome engineering

## Abstract

Cancer stem cells (CSCs), which have the capacity to self-renew and differentiate into various types of cells, are notorious for their roles in tumor initiation, metastasis, and therapy resistance. Thus, underlying mechanisms for their survival provide key insights into developing effective therapeutic strategies. A more recent focus has been on exosomes that play a role in transmitting information between CSCs and non-CSCs, resulting in activating CSCs for cancer progression and modulating their surrounding microenvironment. The field of CSC-derived exosomes (CSCEXs) for different types of cancer is still under exploration. A deeper understanding and further investigation into CSCEXs’ roles in tumorigenicity and the identification of novel exosomal components are necessary for engineering exosomes for the treatment of cancer. Here, we review the features of CSCEXs, including surface markers, cargo, and biological or physiological functions. Further, reports on the immunomodulatory effects of CSCEXs are summarized, and exosome engineering for CSC-targeting is also discussed.

## 1. Introduction

The majority of human cancers display heterogeneity in morphology, expression of cell surface markers, and proliferative or angiogenic potential [1]. During tumor progression, intrinsic mechanisms, including acquired mutations and the “cells of origin”, drive a heterogeneous population of tumor cells, and extrinsic factors from the microenvironment influence the fate of the cells [2]. This results in tumor cells with genetically distinct molecular signatures and therapy resistance [3]. Intravital microscopy studies have shown that tumor cells dynamically interact with their microenvironment, leading to metastasis from the primary tumor [4]. Lineage-retracing experiments in cancer models revealed a subpopulation of cancer cells displaying stem cell-associated characteristics, called cancer stem cells (CSCs), driving tumor growth [5].

CSCs or cancer-initiating cells, which are masked in tumors and have the capacity to self-renew and differentiate into various types of cells, are known to contribute to tumorigenesis [6,7]. Since the CSC concept emerged in the 1990s, it has been one of the most popular cancer research models. The clonal evolution model postulates that malignancies result from the accumulation of genetic instability and sequential selection within the original clone, leading to intra-tumoral heterogeneity [8]. Markers and properties of CSCs have been identified in hematological malignancies, including leukemia, as well as solid tumors, including brain tumors, breast, colorectal, ovarian, pancreatic, and prostate cancers, multiple myeloma, and melanoma [9,10,11,12,13,14]. CSCs exhibit chemo- or radio-resistance, which is attributed to the epithelial–mesenchymal transition (EMT), the signaling pathways, and the DNA damage checkpoint activation, along with the upregulation of CSC markers, including aldehyde dehydrogenase [15,16]. It has been reported that signaling pathways, such as Wnt, transforming growth factor (TGF)-β, Notch, Hedgehog, JAK-STAT (Janus-activated kinase/signal transducer and activator of transcription), and platelet-derived growth factor receptor (PDGFR), are employed by CSCs. Thus, CSCs are highly sought-after as therapeutic targets for the battle against treatment resistance and tumor relapse.

Localized tumor microenvironments as CSC niches have been investigated using 3-D tissue models and microfluidics [17]. Mesenchymal stem cells (MSCs) in the microenvironment secrete proteins, including cytokines and growth factors, which play roles in the differentiation of MSCs [18]. The more recent focus has been on the role of the exosomes secreted from CSCs in modulating CSC niches. Exosomes containing a wide range of RNAs, DNAs, and proteins are released outside of the originating cells through the fusion of multivesicular endosomes or multivesicular bodies (MVBs) with the plasma membrane [19]. The involvement of exosomes in the phenotype transformation from non-CSC to CSC has been recently evidenced by the presence of exosomal FMR (fragile X mental retardation) 1-AS (antisense RNA) 1, the X chromosome long non-coding RNA (lncRNA), which is overexpressed in malignant tumor tissues and activates TLR (Toll-like receptor) 7-NF-κB (nuclear factor kappa-light-chain-enhancer of activated B cells) signaling [20].

Exosomes have been reported to interact with the immune cells modulating the host’s immune response and tumor progression [21]. Tumor-derived exosomes (TEXs) induce apoptosis of the activated cluster of differentiation (CD) 8^+^ T cells, suppress natural killer (NK) cell activity, promote the induction of regulatory T cells (Tregs) and myeloid-derived suppressor cells, and interfere with monocyte differentiation. While TEXs form the immunosuppressive environment, Treg-derived exosomes inhibit the induction of cytotoxic T lymphocytes. Furthermore, exosomes released from the NK cells have shown strong cytotoxicity against tumor cells; this finding has been substantiated by the FasL expressed on the membrane of NK cell-derived exosomes as well as its role in the killing of Fas^+^ tumor cells [22,23]. In addition, a cell-free cancer vaccine candidate using α-fetoprotein-enriched exosomes derived from dendritic cells (DCs) can contribute to adoptive immunotherapy [24]. This stimulates the production of interferon-γ and interleukin (IL)-2 and reduces the expression of TGF-β and IL-10 at the tumor site. The feasibility and safety of DC-derived exosomes and autologous TEXs for the treatment of cancer have been tested in clinical trials [25,26,27,28].

Hence, a deeper understanding and further investigations of the role of CSC-derived exosomes (CSCEXs) in tumorigenicity and the identification of exosomal components could aid in the engineering of exosomes to enhance therapeutic efficacy [29]. The physiological and functional properties of CSCEXs are still under exploration. The immunosuppressive and pro-tumoral capacity of CSCEXs has been studied [30,31]. The CSCEXs induce EMT through the transfer of microRNAs (miRs) to cancer cells and elevate the level of metastasis mediators [32,33]. As cellular expression levels and paracrine or juxtacrine signaling, changed through the transportation of CSCEXs’ miRs into the recipient cells, also contribute to drug resistance, the chemotherapeutic effects, such as cell cycle arrest and apoptosis of cancer cells, can be inhibited by CSCEXs [34,35].

In this review, the features of CSC-associated exosomes, including surface markers, cargo, biological or physiological functions, and immunomodulatory effects, are summarized, and future possibilities for the development of exosome-based cancer immunotherapeutics are discussed.

## 2. Exosomes Derived from Cancer Stem Cells (CSCs)

### 2.1. CSC-Derived Exosomes (CSCEXs)

#### 2.1.1. Expression of CSC Biomarkers in Different Cancer Types

Pancreatic CSCs represent less than 1% of all pancreatic cancer cells and express the surface markers CD44^+^, CD24^+^, and epithelial-specific antigen (ESA)^+^. CD44^+^, CD24^+^, and ESA^+^ CSCs showed robust transcriptional upregulation of the sonic hedgehog and polycomb group gene family member BMI (B cell-specific Moloney murine leukemia virus integration site)-1. Additionally, pancreatic adenocarcinomas contain 1–3% of CD133^+^ cancer cells, some of which also show a high expression of CXC chemokine receptor 4 (CXCR4). These CD133^+^ and CXCR4^+^ cells, but not CD133^+^ or CXCR4 cells, were able to metastasize, and the abrogation of the signaling by CXCR4 similarly blocked tissue invasion. Thus, there may be more than one type of CSC involved in pancreatic adenocarcinomas [36,37].

The presence of CSCs has also been studied in other types of cancer. In hepatocellular carcinomas, the presence of a CD90^+^ subpopulation of tumor cells signifies hepatic CSCs [38]. Colorectal CSCs express CD133^+^, ESA^+^ (EpCAM^+^), CD166, CD44, CD49f, and ESA. CD133^+^ colon CSCs produce IL-4, which is likely to act via CXCR4 to enhance their survival and autocrine growth, which has therapeutic implications [39,40,41]. Integrins a2/b1, B-cell lymphoma (BCL)-2, β-catenin, BMI-1, bromodeoxyuridine (BrdU), Ki67, CD44, CD133, CD49f (integrin a6), CK5/14, CK8/18, glutathione S-transferase (GST)-p, ATP-binding cassette subfamily G member (ABCG) 2/Hoechst 33342, octamer-binding transcription factor (OCT) 3/4, P63, P27, stem cell antigen (SCA)-1, and SMO (Smoothened) are the CSC markers detected in prostate cancers [42,43].

To date, the known representative markers of cancer stem cells (CSCs) comprise CD44, CD24, CD105, and CD133, and additional surface traits are typically tissue-specific [44]. Depending on the cancer type, CSCs have been found to express differential markers in several investigations, and extracellular vesicles (EVs) released from these CSCs share common surface markers [45,46]. Accordingly, recent investigations have reported the tissue-specific surface expression of markers in released CSCEXs.

Brain tumor CSCs were found to express the exosomal markers tumor susceptibility gene (TSG) 101 and flotillin 1, as well as upregulating tenascin-C, resulting in suppressed T cell activity [47]. In hepatic carcinomas, exosomes released from hepatic CSCs were found to exert protumorigenic and prometastatic effects by altering the expression of the targeted molecules p53, BCL-2, vascular endothelial growth factor (VEGF), TGF-β, and matrix metalloproteinase (MMP)-9 [46]. Pancreatic CSCs express upregulated CD44v6 and tetraspanin (TSPAN) 8, and these have been found to release exosomes that contribute to the survival, proliferation, apoptosis, drug resistance, and metastatic potential of pancreatic cancer cells [48,49]. The macrovesicles released from CD105^+^ renal cancer cells were observed to mediate the angiogenic effects, both in vitro and in vivo [50]. Furthermore, exosomes derived from Piwil2-induced CSCs have been found to alter fibroblasts into cancer-associated fibroblasts (CAFs) [51].

#### 2.1.2. CSCEXs and Their Cargo

**Figure 1:**
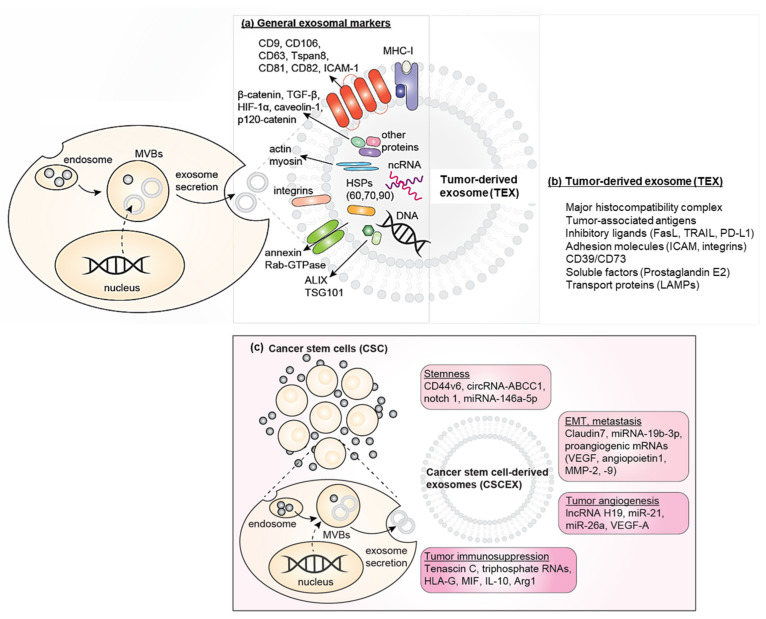
Schematic diagram of cancer stem cell-derived exosomes (CSCEXs) and their cargo. (**a**) General exosomal markers. (**b**) The content unique to tumor-derived exosomes [55]. (**c**) CSCEXs promote cancer stemness, epithelial–mesenchymal transition (EMT)/metastasis, and tumor angiogenesis, and contribute to immunosuppression in the tumor microenvironment [52]. CSCEXs mediate multiple actions through the delivery of microRNA (miR), long non-coding RNA (lncRNA), circular RNA (circRNA), proteins, cytokines, or transcription factors.

EVs released from CSCs play multiple roles, not only by the surface expression of various markers but also by delivering cargo to the receiving cells in the tumor microenvironment (Figure 1) [52]. Exosomes released from CSCs deliver cargo that is thought to be responsible for establishing the pre-metastatic niche, thus contributing to increasing the metastatic potential of CSCs and other associated cells [53,54].

lncRNA H19 was discovered to be secreted through exosomes of CSCs and internalized by surrounding cells that absorb miRs, such as lethal-7 (let-7); this was found to promote the stemness phenotypes of cells surrounding CSCs [56]. A study on breast cancer stem cells showed that released exosomes carry high miR levels associated with metastasis [57], and another study reported that CSC-derived exosomes enhanced cancer cell resistance to chemotherapy, such as doxorubicin and paclitaxel by miR-155 [58]. In colon cancer MSCs, exosomal miR-30a and miR-222 enhanced the tumorigenic phenotype of cancer cells [59]. Analysis of CSCEXs from gastric cancer revealed 11 miRNAs as characteristic features, and this was proposed as a predictive biomarker for metastasis [60]. CSCEXs from gliomas were shown to express high levels of miRNA-21, upregulating VEGF and thus mediating angiogenic effects [61]. Another study reported that hypoxic glioma CSCs contained Linc01060, which activated pro-oncogenic signaling pathways in glioma cells to promote cancer progression [62]. miR-210-3p in lung CSCEXs was found to target the fibroblast growth factor receptor-like 1 (FGFRL1) and promote a pro-metastatic phenotype [63]. Exosomes derived from oral squamous cell carcinoma stem cells were found to express downregulated miR-21 and miR-34, contributing to cancer progression [64]. Furthermore, in gemcitabine-resistant pancreatic CSCs, exosomes were found to express high levels of miR-210, directly transferring the resistant phenotype to receiving cancer cells [34]. In prostate cancer, exosomes from CSCs and non-CSCs have been shown to have different miRNA contents, in which CSCEXs contribute to the premetastatic niche [65]. Similarly, the promotion of metastasis and EMT by CSCEX in renal cell carcinomas was observed by miRNA-19b-3p [32] and was also shown to contain several proangiogenic mRNAs, promoting angiogenesis and the formation of premetastatic niches [50]. Additionally, in papillary thyroid carcinoma, the promotion of EMT was observed through the transfer of lncRNA in papillary thyroid carcinomas [66].

#### 2.1.3. The Current Efforts to Define Differences between CSCEXs and TEXs

Despite the difficulties in distinguishing the CSC population in a tumor lesion, there are current efforts to study the differences between CSCEXs and TEXs. CSCEXs in human prostate cancer were reported to accommodate their unique miRNA content, such as the overexpression of has-miR-1307-5p, compared to exosomes derived from bulk tumor cells [65]. Exosomes secreted by gastric cancer stem-like cells and differentiated cells showed their miRNA expression signature, respectively [60].

Although both CSCEXs and TEXs seem to affect tumor progression, the biomarkers/cargos expressed in CSCs contribute to metastasis more than those expressed in non-stem tumor cells do [67]. Pancreatic CSCEXs transferred the pancreatic CSC marker CD44v6 into non-CSCs, which could reprogram non-CSCs to induce apoptosis resistance, tumor cell motility, and EMT [68]. Interestingly, the injection of colorectal CSCEXs into mice prolonged the viability of neutrophils in the bone marrow, and the neutrophils stimulated with CSCEXs promoted tumorigenesis [69]. Further research is needed to define the differences in biomarkers/cargos and the biological functions between CSCEXs and non-stem TEXs accurately.

## 3. Biological and Physiological Roles of CSCEXs

The complex relationship between CSCs differentiating into cancer cells and their dedifferentiation into CSCs is a dynamic process. As a crucial part of the intermediary cross-talk between all cells within the tumor microenvironment, exosomes are presumed to play a significant role in regulating the balance between CSCs and non-stem cancer cells.

The main function of CSCs is in tumorigenesis and the promotion of cancer cell stemness through the secretion of paracrine factors. Thus, to date, it has been generally speculated that the exosomes derived from CSCs would share this trait and contribute to a pro-tumorigenic microenvironment. Many properties associated with cancer-derived exosomes (CDEXs) overlap with the newly discovered functions of CSCEXs. However, it is important to note that many studies have reported difficulties in distinguishing exosomes released from CSCs from similar subclones within complex tumor tissues [70]. Currently, only a few studies have reported the physiological functions of CSCEXs, which are summarized in relation to the known markers and cargo in the above sections (Table 1).

### 3.1. Role of Exosomes in the Maintenance of Homeostasis between CSCs and Non-Stem Cancer Cells

Homeostasis in the cancer microenvironment is accomplished through the crosstalk between CSCs and non-stem cancer cells, in which the exosomes act as carriers of the important markers required for the regulation of cancer stem cell differentiation and tumor cell dedifferentiation.

Evidently, the exosomes released by stem cell-like breast cancer cells are rich in stem and metastasis-associated mRNA and promote the tumorigenic potential of the recipient cells. The Wnt pathway’s abnormal activation results in tumor development, and the regulation of the self-renewal and differentiation of CSCs, because Wnt signaling plays a major role in growth, development, metabolism, and stem cell maintenance. It has been highly substantiated that fibroblast exosomes activate the Wnt signaling pathway of colorectal cancers (CRCs), allowing CRCs to exhibit stem cell properties, including spherocytosis and tumorigenicity, as well as increasing the proportion of CSCs in CRCs [74,75].

Similarly, exosomes derived from MSCs promote breast cancer cell proliferation by activating the Wnt signaling pathway. In lymphoma, exosomes secreted by collateral cells transport the Wnt signaling pathway in Wnt3a-activated receptor cells, thereby mediating the transformation between the side-population cells and the non-side population cells. However, other signaling pathways have also been activated by exosomes, such as gastric cancer cell-derived exosomes, which promote tumor cell proliferation through the PI3K (phosphoinositide 3-kinase)/Akt (protein kinase B) and MAPK (mitogen-activated protein kinase)/ERK (extracellular-signal-regulated kinase) signaling pathways, and exosomes released from stromal cells that activate the Notch3 signaling pathways in breast cancer cells [75,76].

### 3.2. EMT, Exosomes, and CSCs

The uptake of TEXs into the organ-specific recipient cells induces pre-metastatic niches and the recruitment of bone marrow progenitors [77,78]. The EMT process plays an important role in regulating the self-renewal and differentiation of CSCs, and cells can obtain stem cell phenotypes through EMT processes. TGF-β, which is capable of inducing the onset of EMT, was found in TEXs in recent studies. For instance, exosomes derived from chronic myeloid leukemia transport TGF-β1 abundant in the exosomes to the recipient cells and promote leukemic cell proliferation, colony formation, and tumor formation in vivo. Exosomes released by colon cancer-initiating cells transport cld (claudin) 7 into low metastatic cells, inducing their EMT process [79,80]. CSCEXs were reported to induce EMT in renal cell carcinoma and thyroid cancer via the transfer of miR-19b-3p and lncRNA, respectively [66,81]. Namely, CSCEXs relay CSC secretion and therapy resistance via the transfer of miRs and lncRNAs [82].

### 3.3. Transport of Reprogramming Transcription Factor

Aberrant expression of reprogramming transcription factors in tumor tissues can induce the conversion of non-CSCs to CSCs, and exosomes can regulate the dynamic balance of cancer stem cells by transporting these transcription factors or by regulating the expression levels of the transcription factors in the recipient cells. For example, exosomes secreted by pre-adipocytes promote early breast cancer formation and tumor growth in vivo by transporting the transcription factors SOX (Sry-related high mobility group box) 2 and SOX9 [83,84]. In addition, the miRNAs contained in exosomes also play an essential role in the regulation of tumor cell proliferation, self-renewal, and tumorigenicity. A high expression of miR-222 in melanoma cell-derived exosomes can increase the malignant phenotype of melanoma cells; gastric cancer cells can selectively encapsulate let-7 miRs into exosomes and release them into the tumor microenvironment, thereby promoting the malignant phenotype and tumor growth of gastric cancer. Other oncogenic miRNAs, such as miR-21 and miR-34a, have also been found to be abundant in tumor cell-derived exosomes [85,86].

### 3.4. Immunological Effects of CSCEXs

**Figure 2:**
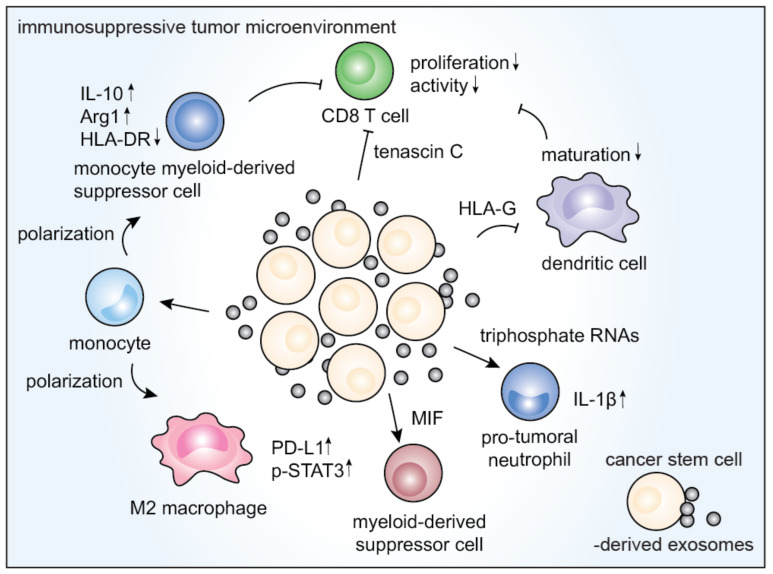
The involvement of CSCEXs in modulating tumor-infiltrating lymphocytes. CSCEXs are known to exhibit the inhibition of proliferation and activation of CD8^+^ T cells, dendritic cell maturation, as well as multiple immunosuppressive effects exerted on M2 macrophages, protumoral monocytes, and neutrophils.

Many studies have revealed that CDEXs or TEXs mediate immunosuppressive functions in the tumor microenvironment [87,88]. Thus, it is logical to presume that CSCEXs share these traits in modulating the tumor immune microenvironment (Figure 2) [89].

Analysis of brain tumor CSCs showed an exosomal release of tenascin-C, which inhibited T cell activation and proliferation [47]. Exosomes from colorectal cancer stem cells have been observed to upregulate interleukin-IL-1β and transform neutrophils into a pro-tumoral phenotype [69]. In another study, colorectal CSC-derived exosomes were loaded onto DCs, which induced CSC-specific T cell responses [90]. Exosomes derived from glioblastoma stem cells were reported to drive the M1 to M2 phenotype transition of monocytes via the STAT-3 pathway, creating an immunosuppressive microenvironment [31].

CSCEXs were also found to contribute to PD-L1 (programmed cell death ligand 1) upregulation in macrophages [31] and to promote the differentiation of peripheral blood monocytes into cells phenotypically similar to M-MDSCs (myeloid-derived suppressor cells) [91]. Furthermore, in glioblastoma CSCs, the secretion of the macrophage migration inhibitory factor (MIF) activates MDSCs and induces immunosuppression [92]. Lastly, in a renal cancer model, CSC-derived EVs mediated the inhibition of DC maturation and T cell-mediated immune responses (Table 2) [93].

There is still much that is unknown regarding CSCEX-mediated immunomodulation in the tumor microenvironment; however, the cross-talk between CSCs and exosomes holds great promise in the development of targeted anti-cancer immunotherapy [95,96].

## 4. Potential for Exploiting CSCEXs

Stem and progenitor cells mediate regenerative properties through paracrine factors, including cytokines and exosomes [30]. The impact of CSCs and CSCEXs on tumor progression can be inferred from the differential expression of several miRNAs in ductal carcinoma in situ stem-like cells. Non-invasive breast CSCs with downregulated miR-140 and upregulated miR-21 and miR-29a show tumorigenicity and a migratory capacity [97,98]. Thus, the specific targeting of RNAs that are aberrantly expressed in CSCs could help in designing CSC-based therapeutics. We can target CSCs using DNA vaccines or mRNA vaccines, but mutagenicity, short half-life, and autoimmunity are the limitations of conventional DNA vaccines or mRNA vaccines [99]. Cell-to-cell transport of exosomal circular noncoding RNAs is involved in the regulation of CSC phenotypes and can influence the tumor microenvironment [100]. So far, the clinical use of circular noncoding RNAs as cancer vaccines has not yet been proven.

### 4.1. Effects of Exosomes on Cancer Stem Cells

Exosomes mediate various effects on CSCs by stimulating the Wnt, Notch, Hippo, Hedgehog, NF-κB, and TGF-β pathways, among others [101]. It follows that exosomes can exert numerous downstream effects involving differentiation, tumorigenesis, and other crucial endogenous functions of CSCs [102].

In breast cancer, CD44^−^ cell proportions were observed to increase from CD44^high^/CD24^low^ CSCs through cell cycle inhibitory miRNA delivery by exosomes [103]. Moreover, in ovarian cancer, the exosomal release of miRNA-454 was observed to sustain the stemness phenotype of cancer cells [104]. Another example of prostate CDEXs with the surface expression of caveolin-1 was observed to transform CSCs into a metastatic phenotype via NF-κB signaling [105]. Furthermore, the interactions of CSCs with CAFs have been observed by using CAF-derived exosomes and their miRNA content, such as miR-21, miR-378e, and miR-143 [53]. These were also observed to promote tumor progression, except for miR-320, which antagonized the premetastatic niche formation [53].

Recent studies have proposed superior tumor growth inhibitory effects by targeting CSCs rather than the whole tumor [106]. Thus, potential strategies for targeting CSCs by delivering miRNAs to inhibit the EMT or the formation of premetastatic niches have been thoroughly researched [107]. Neutralizing antibodies, or antibody-mediated CSC therapies, aim to target CSCs in various cancers to attenuate the stemness phenotype of these cells [107]. Exosome signaling is also known to induce the production of CSCs, ameliorate treatment resistance, and prevent tumor relapse [108]. There is also a growing interest in exosome engineering to target specific signaling pathways by miR or siRNA inhibitors for CSC modulation [101].

### 4.2. Targeting Cancer Stem Cells through Engineered Exosomes

The identification of various potential markers of CSCs expressed in different cancer types provides a possible means for targeting CSCs specifically using engineered exosomes. Targeting the CD44 expressed in hepatocellular carcinoma in metastatic hepatocellular CSCs using anti-CD44 antibody-coated exosomes could directly induce CSC death. Likewise, the CSC markers of different cancers, such as CD24^+^, CD133, CD90^+^, ESA^+^ (EpCAM^+^), CD166, CD44, CD49f, integrins a2/b1, BCL-2, β-catenin, BMI-1, BrdU, Ki67, CD44, CD133, CD49f (integrin a6), CK5/14, CK8/18, GST-p, ABCG2/Hoechst 33342, OCT3/4, P63, P27, SCA-1, SMO, and CD200, can be targeted using engineered anti-antibody-coated exosomes. In addition, exosomes engineered for targeting different signaling pathways that contribute to self-renewal, differentiation, tumor initiation, and drug resistance in CSCs could aid in the effective design of engineered exosomes for cancer treatment [109,110].

According to the differential roles of surface markers/cargo in exosomes derived from CSCs and non-stem cells, exosomes engineered with surface markers/cargo unique to CSCs are expected to have potential for the treatment of cancer. CSCEXs contain multiple stemness marker proteins, such as CD44v6 and Notch1, which generate transient or dynamic tumor heterogeneity in the tumor microenvironment compared to non-cancer exosomes [73]. CSCEXs’ RNA cargo, such as miR-19b-3p, playing a unique role in metastasis can also be envisaged as a therapeutic target. The exosomal miR-210-3p isolated from lung CSCs contributed to the pro-metastatic niche of lung cancer, while miR-210-3p in non-stem TEXs mainly promoted tumor angiogenesis. Triphosphate RNAs unique to CSCEXs, which facilitate the pro-tumoral phenotype of neutrophils, can be targeted for cancer immunotherapy [69].

## 5. Conclusions and Future Directions

CSCs, or cancer-initiating cells, are tumorigenic and give rise to local and distal tumor recurrence. A subpopulation of cancer cells possessing self-renewing and multipotent properties is a potent hindrance to conventional cancer therapies that solely target the existing malignant cells. Many investigations have focused on gaining insights into the biological properties of CSCs and their secretions for the development of novel therapeutic interventions specifically targeting CSCs. Recent investigations have established the significance of exosomes in cell-to-cell communication and the formation of a unique niche for the homeostasis of CSCs and non-stem cancer cells; this makes CSCs an ideal target in disrupting this balance. We can focus on targeting and exploiting Evs, including the exosomes released from CSCs, as a potential strategy for eliminating CSCs [111].

Further efforts are needed to elucidate the complex biological effects of CSCEXs on tumorigenesis, metastasis, and cancer immunity. Thus, a better and more thorough understanding of the characteristics and contents of these CSCEXs and other EVs may give way to the development of new clinical diagnostic/prognostic tools and therapies to prevent tumor resistance and relapse [112].

## Figures and Tables

**Table 1:** Functions of surface markers/cargo found in tissue-specific cancer stem cell-derived exosomes (CSCEXs).

Source	Surface Marker/Cargo	Effect	Reference
Breast CSCs	miR-155	Enhanced resistance to doxorubicin, paclitaxel	[58]
Colorectal CSCs	miR-30a, miR-222	Promotion of tumorigenicity through targeting MIA-3	[59]
	Triphosphate RNAs	Promotion of tumor phenotype of neutrophils	[69]
	circRNA-ABCC1	Promotion of cancer stemness	[71]
Gastric CSCs	hsa-miR-1290, -1246, -21-5p, -100-5p, -20a-5p, -26a-5p, -24-3p, -182-5p, -378a-3p, -148a-3p, -17-5p, -23a-5p; has-let-7f-5p, -7a-5p, -7g-5p	Predictive biomarkers for metastasis	[60]
	Claudin7	Promotion of metastasis	[72]
Glioblastoma CSCs	Tenascin C	Inhibition of T cell proliferation and activation	[47]
	Linc01060	Promotion of cancer progression by activation of pro-oncogenic signaling	[62]
	Notch1	Promotion of cancer stemness	[73]
Lung CSCs	miR-210-3p	Pro-metastatic phenotype in lung cancer cells by targeting the fibroblast growth factor receptor-like 1 (FGFRL1)	[63]
Oral squamous cell carcinoma CSCs	Downregulation of miR-34	Cancer progression	[64]
Pancreatic CSCs	miR-210	Gemcitabine-resistant phenotype transference to pancreatic cancer cells	[34]
	CD44v6	Promotion of cancer stemness and metastasis	[68]
Prostate CSCs	Hsa-miR-1307-5p, -139-5p, -148a-3p, -183-5p	Contribution to the premetastatic niche	[65]
Renal cell CSCs	Proangiogenic mRNAs (VEGF, angiopoietin1, MMP-2, MMP-9)	Angiogenesis and promotion of premetastatic niche formation	[50]
	miR-19b-3p	Promotion of EMT	[32]
Papillary carcinoma CSCs	lncRNA DOCK9-AS2	Promotion of proliferation, migration, and invasion by activation of Wnt/β-catenin pathway	[66]

CSC: cancer stem cell; CSCEX: cancer stem cell-derived exosome; miR: microRNA; MIA: melanoma inhibitory activity; circRNA: circular RNA; has: homo sapiens; LINC: long intergenic non-protein coding RNA; VEGF: vascular endothelial growth factor; FGFR1: fibroblast growth factor receptor-1; MMP: matrix metalloproteinase; EMT: epithelial-to-mesenchymal transition; lnc: long noncoding; AS2: antisense RNA2.

**Table 2:** Immunological properties of cancer stem cell-derived exosomes (CSCEXs).

Source	Surface Marker/Cargo	Immunological Effects	Reference
Colorectal CSCs	Triphosphate RNAs	Neutrophil transformation into pro-tumoral phenotypes, interleukin (IL)-1β secretion	[69]
	CSCEX loaded onto dendritic cells (DCs)	Activation of CSC-specific T cell response	[90]
	miR-146a	Increase in tumor-infiltrating CD66^+^ neutrophils, decrease in tumor-infiltrating CD8^+^ T cells	[94]
Glioblastoma CSCs	Tenascin-C	Inhibition of T cell activation, proliferation	[47]
	Eukaryotic initiation factor 2, mTOR, and ephrin B signaling pathways	M1 to M2 transition of monocytes via the STAT-3 pathway, upregulation of PD-L1 in macrophages	[31]
	Increased IL-10 and Arg1, downregulated MHC class II cell surface receptor (HLA-DR)	Differentiation of peripheral blood monocytes into cells similar to M-MDSCs	[91]
	MIF	Activation of MDSCs	[92]
Renal CSCs	HLA-G	Impairment of DC maturation and T cell-mediated immune response	[93]

CSC: cancer stem cell; CSCEX: cancer stem cell-derived exosome; IL: interleukin; DC: dendritic cell; mTOR: mechanistic target of rapamycin; Arg1: gene encoding the protein arginase; MHC: major histocompatibility; HLA: human leukocyte antigen; MIF: gene encoding macrophage migration inhibitory factor; STAT: signal transducer and activator of transcription; PD-L1: programmed cell death ligand 1; MDSC: myeloid-derived suppressor cell.

## Data Availability

Not Applicable.
